# Emulsion-Templated Liquid Oil Structuring with Egg White Protein Microgel- Xanthan Gum

**DOI:** 10.3390/foods12091884

**Published:** 2023-05-03

**Authors:** Yujie Su, Wanqiu Zhang, Ruidan Liu, Cuihua Chang, Junhua Li, Wen Xiong, Yanjun Yang, Luping Gu

**Affiliations:** 1 State Key Laboratory of Food Science and Technology, Jiangnan University, Wuxi 214122, China; suyujie@jiangnan.edu.cn (Y.S.);; 2 School of Food Science and Technology, Jiangnan University, Wuxi 214122, China; 3 Hunan Engineering & Technology Research Center for Food Flavors and Flavorings, Jinshi 415400, China

**Keywords:** egg white protein, microgel, xanthan gum, emulsion-template method, oleogels

## Abstract

In this study, oleogels were prepared by the emulsion-template method using egg-white protein microgel as a gelator and xanthan gum (XG) as thickener. The physicochemical properties of the emulsion and oleogels were investigated. The adsorption of protein on the surface of the oil droplet reached saturation when the protein microgel concentration reached 2%. The excess protein combined with XG and accumulated on the outer layer of the oleogel, which prevented the emulsion from flocculation, enhanced the oil-holding capacity of the oleogel, and had a positive effect on preventing the oxidation of oil. When the concentration of XG was less than 0.4%, the EWP microgel, combined with the XG, stabilized the emulsion. As the concentration of XG was greater than 0.4%, excessive XG in the emulsion improved the viscosity and mechanical properties of the emulsion to prevent the aggregation of oil droplets. However, the change in XG concentration had no significant effect on the oxidation of the oil.

## 1. Introduction

Solid fats play a unique role in the sensory properties of food, while trans fatty acids and saturated fatty acids in solid fats pose a risk to human health. A large intake of trans fatty acids and saturated fatty acids in the diet will greatly increase the risk of cardiovascular diseases, so it is reasonable to replace saturated fatty acids with unsaturated fatty acids [1,2]. However, solid fats can provide foods with specific structural properties (such as consistency, expansion, hardness, and brittleness) and sensory properties (such as taste, appearance, smell, and touch), that are not achieved by liquid oils low in saturated and trans fats [3]. As a result, researchers have focused on developing a way to replace solid fats from a health and sensory perspective. The preparation of oleogels from liquid oils has received widespread attention as a potential approach in recent years.

Oleogels have the composition of liquid oils but show similar physicochemical properties to solid oils. An oleogel is a gel-like oil with a certain viscoelasticity and high oil content (generally greater than 90%), composed of liquid oil and a small amount of gelators (structuring agents) [4]. Unlike traditional solid fats, which are three-dimensional network structures formed by triglyceride crystals, the aggregates formed by the gelators then entangle with each other to create a three-dimensional network structure [5]. Oil structuring agents can be divided into two major groups, low and high molecular weight oil gelators (LMOG and HMOG). LMOGs that have been shown to form oleogels are monacylglycerides, phospholipids, plant and animal waxes, shellac, sorbitol esters, etc., [6]. Generally, HMOGs are polymers, such as protein, ethyl cellulose, hydroxypropyl methyl cellulose, methyl cellulose, chitosan, chitin, etc. Among them, proteins are food-grade, nutritive, and environmentally friendly. They have great advantages in the structuring of oleogels and are accepted by consumers [7]. However, proteins as water-soluble polymers cannot be dissolved in vegetable oil directly. Therefore, oleogels structured by proteins need to be prepared through indirect methods, such as the emulsion-template method [8]. Oleogels are prepared in three steps according to the emulsion-template method. First, emulsions with high oil content are prepared through certain treatment methods, and the interaction between droplets or polymers leads to a network structure. Then, the water contained in the emulsion is removed after a drying treatment. Finally, oleogels can be obtained after shearing [9]. The protein can stabilize the emulsions and thus the oleogels.

Many types of proteins have been studied to prepare oleogels by the emulsion-template method, including gelatin [10,11,12], β-lactoglobulin [13], sodium caseinate [14], soy protein isolate [15,16], and whey protein isolate [17,18,19]. Patel et al. [10,11] reported oleogels with an oil content of up to 97% using the template prepared with gelatin as a gelator, and the oil droplets in oleogels were found to be tightly packed together with a distinct layer of polymers preventing the coalescence of oil droplets. The formation of stable oleogels might benefit from the establishment of a stable, thick protein-interface layer [8,15]. Egg-white protein (EWP) has a complete nutritional profile and multiple functional properties, such as emulsification, gelation, and foaming, and is widely used in the food industry [20]. EWP may be an appropriate and potential natural matrix for formulating oleogels. However, few studies have reported the preparation of emulsion-template oleogels with EWP. The possible reason for this is that EWP cannot play a role as a good emulsifier under neutral conditions because most hydrophobic amino acids are embedded in the native state, leading to strong hydrophilicity and the poor physical stability of emulsions [21,22]. Recently, some studies have proved that microgels prepared from EWP are good stabilizers of Pickering emulsions and foams, which might be due to the production of interfacial films with high elasticity and desorption energies [22,23,24]. The success of the emulsion-template approach is dependent on the formation of a stable interfacial layer, which is hard enough to withstand dehydration [4]. Therefore, it seems feasible to use the interfacial behavior of EWP microgels to prepare emulsions and oleogels. Meanwhile, microgels and protein particles have been reported as a means of replacing native proteins to stabilize emulsions and achieve oil structuring [25,26].

Proteins alone can stabilize the interface but are more often to be used with polysaccharides to increase stability [27]. Polysaccharides may be situated at the interface, together with the protein due to protein–polysaccharide interactions or increase the stability of oleogels by enhancing the viscosity of the continuous phase and preventing oil droplet coalescence [8,28]. Xanthan gum (XG) is one of the most common polysaccharides used as a thickening agent to prepare emulsion-template oleogels [10,14,17,29]. Espert et al. [17] obtained stable and solid-like oleogels structured by XG in combination with four different structuring agents (a synthetic one, a protein, a lipid, and a polysaccharide). XG is an anionic polysaccharide with good stability, thickening, suspension, and safety and is widely used in the food, cosmetic, and medicine fields. XG fully swells in water to form a network structure, which limits the flow of water and thus shows good thickening properties. Due to the obvious thickening effect and weak gel structure, XG can increase the continuous phase viscosity and improve the physical stability of the emulsion [14,30,31]. EWP microgels may have a good effect on the stability of emulsion and oleogels, and XG is also widely used in the preparation of oleogels, but there are few studies on the methods and effects of the two together in the preparation of oleogels. We hypothesized that the EWP microgel and XG could stabilize the emulsion and the oleogels well, which would further expand the application of EWP for the preparation of oleogels.

In this study, the emulsion-template method was adopted to prepare the oleogels using EWP microgels as a gelator and XG as a thickener. The objective of the study was to investigate the effects of EWP microgel and XG concentrations on the properties of emulsions and oleogels. The properties of the emulsions and physicochemical properties of the oleogels were determined to study the individual and combined roles of EWP microgels and XG. The results may provide a new approach for the preparation and application of oleogels.

## 2. Materials and Methods

### 2.1. Materials

The EWP powder used in this study was provided by Jiangsu Kangde Egg Industry Co., Ltd. (Nantong, China). Sunflower oil was purchased from a local supermarket in Wuxi, China. 1,1,3,3-Tetraethoxypropane was purchased from Shanghai Macleans Biochemical Technology Co., Ltd. (Shanghai, China). XG was purchased from Sigma-Aldrich, Co., Ltd. (St. Louis, MO, USA). Nile red, Rhodamine B, barium chloride, ammonium thiocyanate, isopropylbenzene hydrogen peroxide, ferrous sulfate, trichloroacetic acid, 2-thiobarbituric acid and other chemical reagents were purchased from Sinopharm Chemical Reagent Co., Ltd. (Shanghai, China). All chemical reagents were of analytical grade.

### 2.2. Preparation of Aqueous Dispersions

EWP microgels were prepared following an earlier technique with some modifications [22,32]. The EWP powder was dissolved in deionized water at 4 wt%, and the pH was adjusted to 7.0 with 1.0 M HCl. The resulting EWP solution was heated at 90 °C for 30 min in a thermostatic water bath (HWS24, Yiheng, Shanghai, China), and then immediately placed at 4 °C to form a gel. The gel was disintegrated with a high-speed shear disperser (IKA T25 Basic, Staufen, Germany) at 11,000 rpm for 4 min and then refrigerated at 4 °C until further use. The XG powder was dissolved in deionized water to form a dispersion at a concentration between 0.1 wt% and 1.6 wt%, which was refrigerated at 4 °C for later use.

### 2.3. Preparation of Emulsion-Templated Oleogels

Crude emulsions were prepared by mixing the EWP microgels and sunflower oil with a high-speed shear disperser at 11,000 rpm for 4 min. After that, an appropriate amount of XG dispersion was added into the crude emulsion and mixed with the same shear disperser at 11,000 rpm for 4 min to prepare emulsions (almost 100 g). The emulsions were tiled in a glass plate and dried in an oven (DGG-9140A, Senxin, Shanghai, China) at 60 °C to a constant weight (about 24 h). Finally, the oleogels were obtained by appropriate manual shearing in order to make the emulsions homogeneous.

To study the effects of the protein concentration on the properties of emulsions and oleogels, the emulsions were prepared at EWP microgel concentrations of 0, 0.5, 1, 2, 3 and 4% by diluting the microgels of 4 wt%, while the concentration of XG in its dispersion was kept at 1.2%. The prepared emulsions contained 55 wt% EWP microgel dispersion, 30 wt% sunflower oil and 15 wt% of the XG solution. The effect of the XG concentration was assessed by adjusting the concentration of XG in its dispersion to 0, 0.1, 0.4, 0.8, 1.2 and 1.6% while keeping the EWP microgels concentration at 4%. This set of emulsions contained 27.5 wt% of the EWP microgel dispersion, 30 wt% sunflower oil, and 42.5 wt% of XG dispersion. A clear description of the experimental design is shown in Table 1 and Table 2.

### 2.4. Emulsion Characterization

#### 2.4.1. Determination of Physical Stability and Droplet Size

The emulsion was diluted two-fold with deionized water, and was then added to a small sample bottle, and the sample was left standing at 25 °C for 48 h to observe the phase separation of the sample.

A laser particle size analyzer (S3500, Microtrac Inc., Largo, FL, USA) was used to determine the droplet size of the emulsion after preparation. The mean droplet size was reported for the volume-weighted average diameter (d_4,3_). All experiments were conducted at 25 °C, and the results were averaged among the three experiments.

#### 2.4.2. Microstructure of the Emulsions

The microstructure of the emulsion was observed with an Axiolab A reflected light-microscope (Zeiss, Berlin, Germany) equipped with a Power Shot G2 photographic camera (Canon, Tokyo, Japan) under a 10× objective lens. After pipetting 10 µL of an emulsion sample on a glass slide and covering with a cover glass, micrographs were taken after incubation for 2 min.

#### 2.4.3. Rheological characterization of the emulsions

Rheological characterization was performed using a rheometer (DHR-3, Waters, MA, USA). The measurement was performed by a 40 mm parallel plate with 1000 µm plate gap at 25 °C. Shear rate ramps were conducted from 1 s^−1^ to 100 s^−1^. In the dynamic oscillation test, strain sweep tests were carried out at 1 Hz and a strain range of 0.1~100% to determine the limit of linear viscoelastic regime (LVR). Frequency sweep tests were then conducted at 1 Hz and 1% strain.

### 2.5. Characterization of Oleogels and Dried Products

#### 2.5.1. Microstructure of the Dried Products

The microstructure of dried samples was observed with a confocal laser scanning microscope (LSM 710, Zeiss, Berlin, Germany) under a 20× objective lens. By adding proteoglycan stain—Rhodamine B and grease stain—Nile red together into the emulsion for dyeing and filming, then the slides were dried in a 60 °C constant temperature oven to constant weight for observation.

#### 2.5.2. Determination of Oil Loss Rate of the Oleogels

According to the methods of Kanagaratnam et al. [33] with some changes, the oleogel was placed on filter paper for 24 h after centrifugation. Oil loss rate (OL%) was calculated according to Equation (1):

OL(%) = (m_1_ − m_2_)/m_1_ × 100% (1)

where m_1_ is the mass of oleogels (g) before 24 h, and m_2_ is the mass of oleogels (g) after 24 h.

#### 2.5.3. Determination of Oxidation Stability of the Oleogels

The peroxide value (POV) of the oleogels was determined according to a previous method [34] with a slight modification. After adding 0.5 g of oleogels into 1.5 mL of isooctane/isopropanol (3:1, *v/v*) mixture, the mixture was centrifuged at 2500 rpm for 3 min. The 200 µL of the supernatant sample was mixed with 5.6 mL of methanol/n-butanol (2:1, *v/v*), 200 µL of distilled water, 30 µL of 3.94 M ammonium thiocyanate solution, and 30 µL of Fe^2+^ solution (a mixture with 0.144 M FeSO_4_ and 0.153 M BaCl_2_) in sequence. After the mixture was evenly mixed and placed at 25 °C for 20 min, the absorbance value of the reaction solution was measured at 510 nm (UV-2600 UV-Visible Spectrophotometer, Hitachi, Tokyo, Japan). A standard curve was prepared with cumene hydroperoxide in absolute ethanol to calculate the POV value of the oleogels according to Equation (2):

POV(mmol/kg) = (A − 0.0026) × 10/2.1232 m(2)

where m is the mass of oleogels (g), and A is the absorbance value (510 nm).

The thiobarbituric acid reactive substances (TBARS) of the oleogels was determined in accordance with the method of Zhao et al. [35] with a slight modification. The 0.5 g of oleogels was added into 3 mL of trichloroacetic acid (15%, *w/v*)/thiobarbituric acid (0.375%, *w/v*) solution, which was mixed with a vortex mixer and heated in a boiling water bath for 30 min. After cooling to room temperature, the solution was centrifuged for 5 min at 6000 rpm. The supernatant was then filtered with a 0.22 µm PES membrane, and the absorbance of the filtrate was measured at 532 nm. The TBARS value of the oleogels was calculated according to Equation (3):

TBARS(mg/kg) = (A − 0.0045)/0.3406 m(3)

where m is the mass of oleogels (g), and A is the absorbance value at 532 nm.

### 2.6. Statistical Analysis

All samples were prepared three times and all the experiments were carried out three times. Statistical analyses were performed using the statistical program SPSS (SPSS Inc., Chicago, IL, USA) and Origin Pro 9.0 (OriginLab, Northampton, MA, USA). The results are reported by means and standard deviations. The difference between the means was evaluated using Duncan’s test (*p* < 0.05).

## 3. Results and Discussion

To simplify descriptions of the treatments, the EWP microgels and XG concentrations in their dispersions used to prepare emulsions are used hereafter. These concentrations are not their overall compositions in the entire emulsion or oleogel.

### 3.1. Characterization of Emulsions

#### 3.1.1. Macro-, Micro-Structure, and Droplet Size of Emulsions

The properties of the emulsion that could affect the performance of the oleogels—the macro- and micro-structure of the emulsions and the d_4,3_ of droplets—were analyzed.

**Figure 1:**
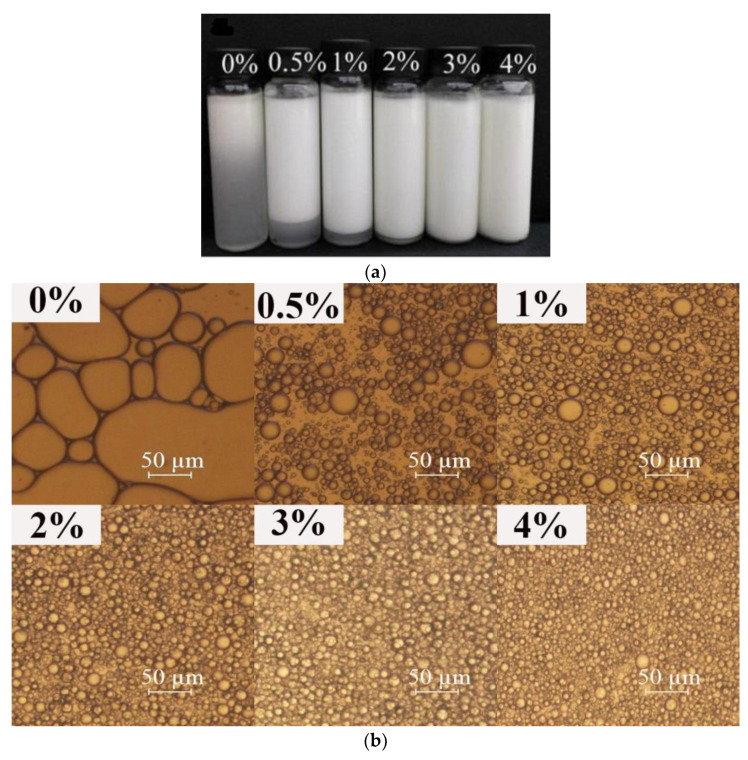

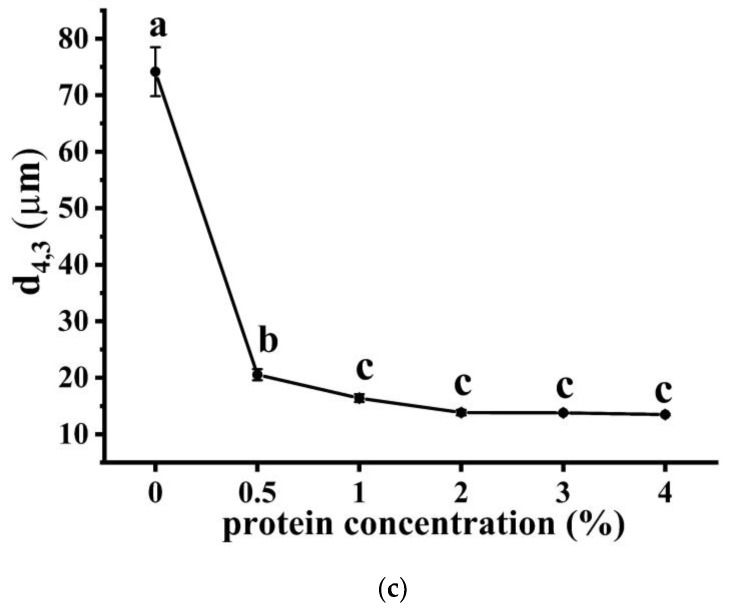
The effect of EWP concentration on macrostructure (**a**); microstructure; (**b**) and droplet size (**c**) of emulsions. The error bars are the standard deviation of three repetitions. Different lowercase letters show significant differences (*p* < 0.05).

**Figure 2:**
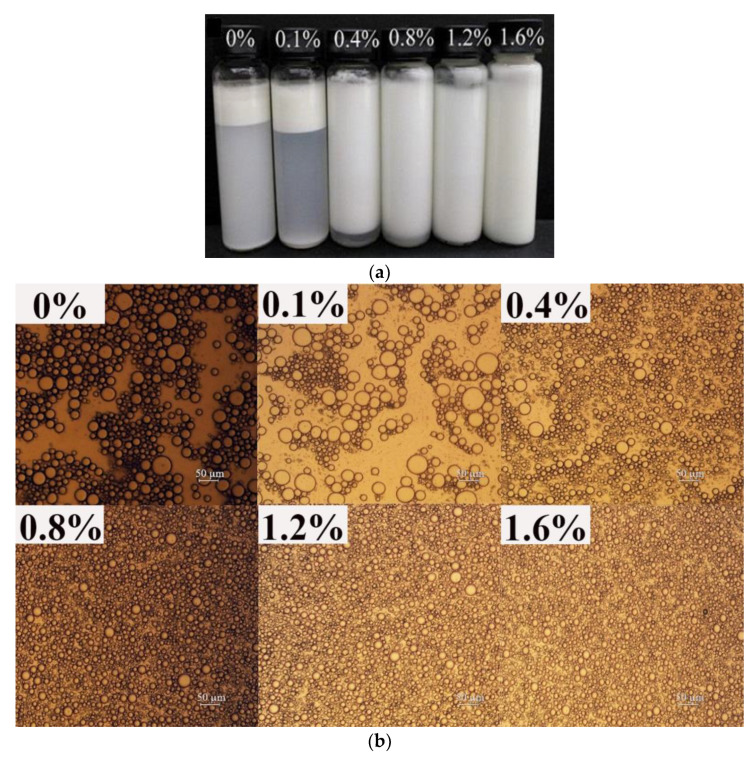

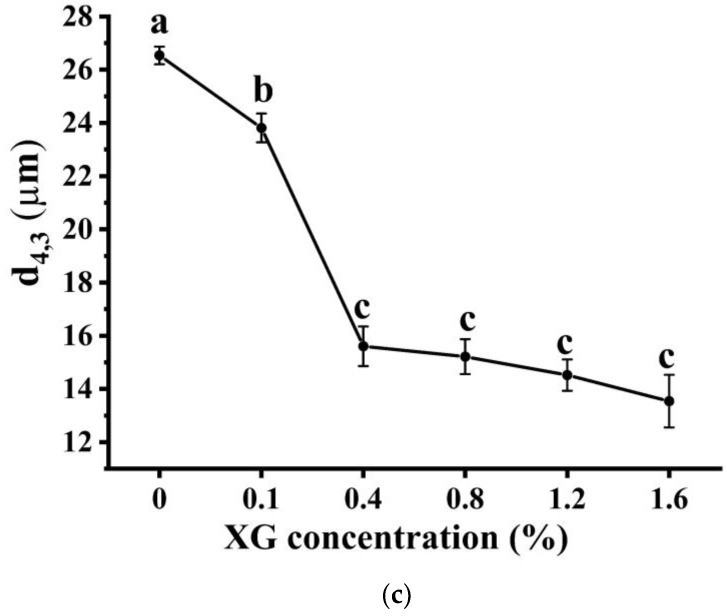
The effect of XG concentration on macrostructure (**a**); microstructure (**b**); and particle size (**c**) of emulsions. The error bars are the standard deviation of three repetitions. Different lowercase letters show significant differences (*p* < 0.05).

Figure 1 and Figure 2 show the influence of the concentration of EWP microgels and XG on the properties of emulsions, respectively. As shown in Figure 1a, the flocculation of emulsions gradually weakened with the increase of the EWP microgel concentration. When the EWP microgel concentration reached 3%, the emulsion did not show obvious phase separation. Figure 1b shows that the emulsion droplets became smaller with the gradual increase of EWP microgel concentration, which was similar to previous research [11,36]. When the concentration of EWP microgels was higher than 2%, the size of the emulsion droplets did not seem to change with the further increase of the EWP microgel concentration. In Figure 1c, the d_4,3_ decreased from 74 µm to about 16 µm when the concentration of EWP microgels increased from 0% to 1%. When the concentration of EWP microgels increased from 1% to 4%, the d_4,3_ decreased from 16.41 µm to 13.5 µm; however, the difference was not significant. (*p* > 0.05). This was expected because the EWP microgels are surface-active [37,38] and a greater amount of the microgels results in an increased availability to adsorb on oil droplets during emulsification and form stronger interfacial films [24]. As the concentration of the protein microgels increased, the amount of microgels adsorbed on the surface of the oil droplets increased, gradually forming a layer of protein film, which effectively inhibited the aggregation of the oil droplets, and the droplet size became smaller gradually. However, when the surface of the oil droplets was completely absorbed by the protein microgels, the protein on the surface of the oil droplets reached adsorption saturation, the excessive protein microgels continued to surround the outer layer of the droplets, and the droplet size of the emulsion no longer decreased with the increase of the protein microgel concentration.

Similarly, as shown in Figure 2, the addition of XG improved the stability of the emulsion. As can be seen from Figure 2b,c, with the increase in the concentration of XG, the emulsion droplets became smaller gradually and the distribution of droplets became more uniform. When the XG concentration was greater than 0.4%, the particle size of the emulsion droplets decreased slowly, and even experienced no changes. Patel, et al. [11] reported that the change of XG concentration from 0.6 to 1.5% at a fixed gelatin concentration did not show any prominent change in the average volume mean diameter of an emulsion. As shown in Figure 2a, when the concentration of XG was higher than 1.2%, the emulsion did not have obvious phase separation. XG has good thickening properties and increasing the concentration of XG can effectively increase the viscosity of the system, thus playing the role of stabilizing the emulsion. These results were consistent with the results obtained by Sun, et al. [39] and Chityala, et al. [40], where the increased viscosity restricted droplet movement and reduced droplet aggregation [41]. The addition of anionic polysaccharide XG also improved the electrostatic repulsion between the oil globules, so the stability of the emulsions was enhanced accordingly [42].

#### 3.1.2. Rheology of Emulsions

**Figure 3:**
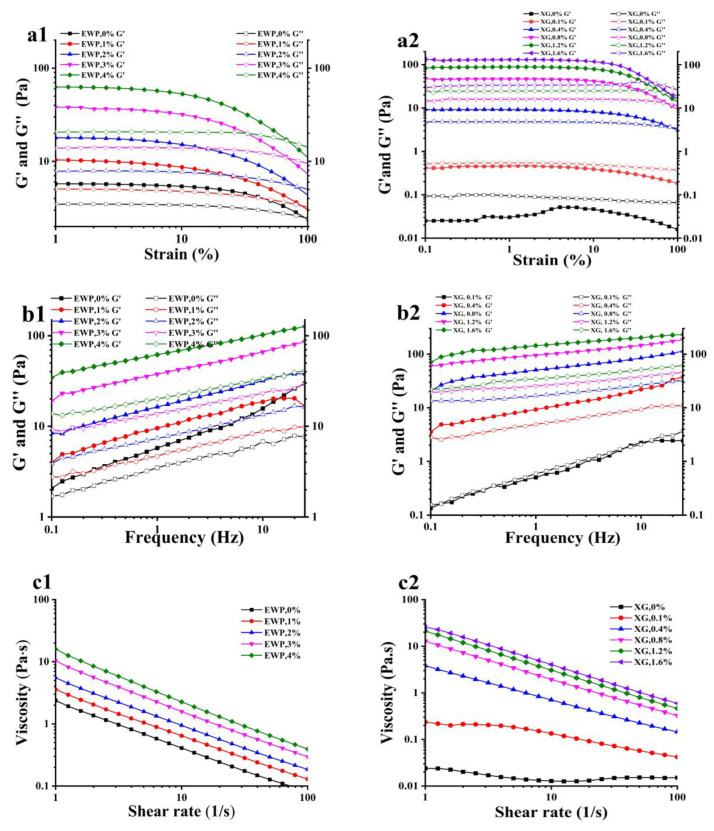
Effects of EWP concentration (**a1**,**b1**,**c1**) and XG concentration (**a2**,**b2**,**c2**) on rheological behavior of emulsions at 25 °C: (**a**). strain-sweep curves; (**b**) frequency-sweep curves; and (**c**) shear-rate sweep curves.

The rheological properties of the emulsions were characterized using small amplitude oscillatory measurements, which have important influences on the processing and stability of emulsions, and also then affect the properties of oleogels. (Figure 3). As the preliminary experiment results showed that there was little difference between rheological results at 0.5% and 1% protein concentration, samples with 0.5% protein concentration were not selected for this part of the experiment. Strain sweep tests were used to determine the linear viscoelastic region (LVR) of the emulsions (Figure 3(a1,a2)). It can be seen from the frequency scanning curves of emulsions with varying EWP microgels concentrations (Figure 3(b1)) that the G’ of all emulsions were greater than G’’, and the emulsions behaved as solid properties, indicating that elastic gelatinous emulsions were formed under these conditions [43]. In the presence of XG, an increase of the EWP microgel concentration brought about a progressive increase in the gel strength, as seen from an increase in the G’, which can be caused by the strength of the network formed by the EWP in the bulk phase and at the interface [11]. However, as shown in Figure 3(b2), when the concentration of XG was 0.1% while keeping EWP microgels concentration at the same, the G’ of the emulsions were less than G”, which showed the liquid properties. When the concentration of XG was greater than 0.4%, G’ was greater than G”, which showed the emulsions displayed a “gel-like” behavior. Therefore, the addition of XG gradually increased the gel viscoelasticity of the emulsions. As mentioned above, the particle size of the emulsion droplets decreased with an increase in the XG concentration.

In Figure 3(b1), within the low-frequency scanning range (0.1~10 Hz), G’ of all emulsions was greater than G” and increased with the increase in frequency; that is, G’ is frequency-dependent. However, in the experiment to explore the XG concentration, it is worth noting that when the concentration of XG was less than 0.4%, the G’ increased significantly with the increase in frequency. When the concentration of XG was in the range of 0.8~1.6%, the dependence of G’ on frequency was not obvious (Figure 3(b2)). The strong dependence of G’ on frequency indicated that the gel strength of the emulsions was weak [28], as described in Figure 3(b1). The results showed that the gel strength of the emulsion was improved by adding XG, and that the high concentration of XG (0.8~1.6%) showed no further improvements. It may be because the amount of XG added reached saturation when the mechanical strength of the emulsions was highest. Therefore, when the XG concentration was between 0.8–1.6%, the particle size of the emulsion was smaller, although this was not significant.

As shown in Figure 3(c1,c2), the viscosity of most emulsions decreased with the increasing shear rate, showing a typical shear-thinning behavior [42]. This might be because the emulsion droplets were close enough and interacted to form a network. As the shear rate increased, the network was broken, resulting in a reduction in viscosity [44]. It is worth noting that when the concentration of XG was 0%, the apparent viscosity of the emulsions stabilized by EWP microgels alone remained almost unchanged with the increase in shear rate, while the emulsions with the use of XG exhibited shear-thinning behavior (Figure 3(c2)). This may have been due to the network formed by the XG molecules, which may lead to the shear-thinning behavior of the emulsion [45]. The flow resistance was relatively large because of the entanglement and flocculation between protein and XG molecules at a low shear rate, and the shear stress was not high enough to destroy the interaction between the systems. With the increase in shear rate, the emulsion flowed along the direction of shear under the action of shear force, and the resistance decreased accordingly. Moreover, when the protein microgel concentration was 0%, the emulsions with XG alone also exhibited shear-thinning behavior (Figure 3(c1)), which may have been because the XG solution is a pseudoplastic fluid with a shear-thinning phenomenon [17].

### 3.2. Characterization of Oleogels and Dried Products

#### 3.2.1. Microstructure of Dried Products

**Figure 4:**
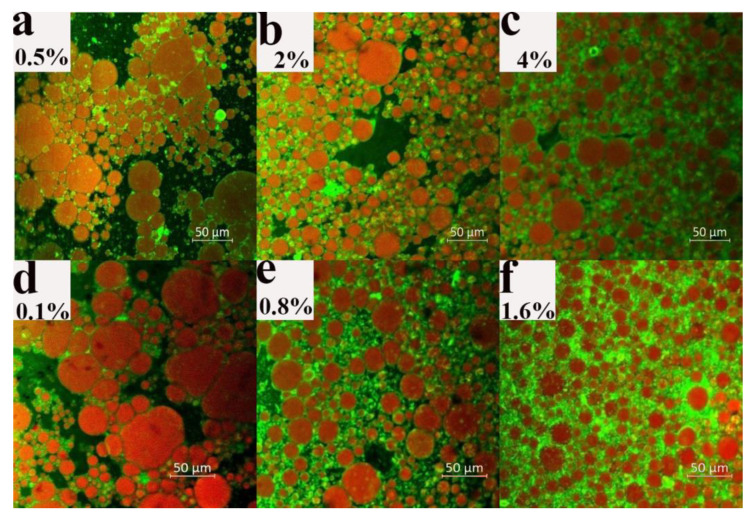
Effects of EWP and XG concentrations on the microstructure of oleogels. The EWP concentration in its dispersion was: (**a**) 0.5%; (**b**) 2%; and (**c**) 4%. The XG concentration in its dispersion was: (**d**) 0.1%; (**e**) 0.8%; and (**f**) 1.6%.

**Figure A1:**
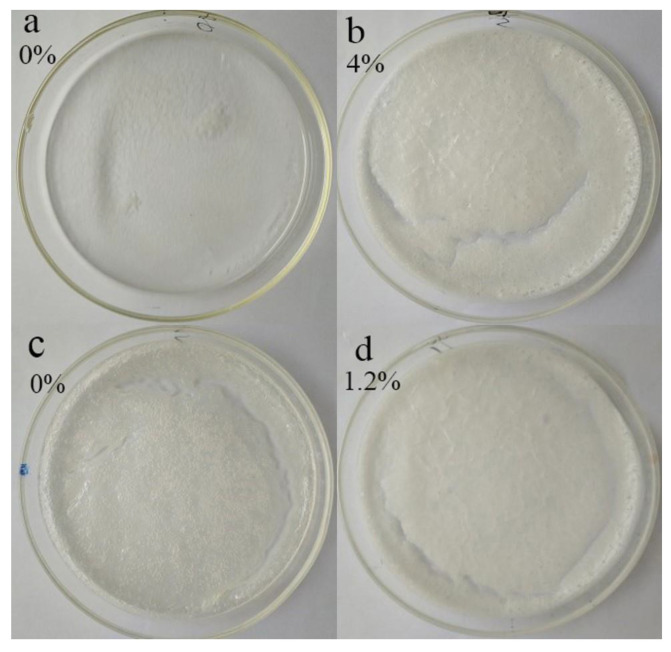
Effect of protein microgel and XG concentration on macro-pictures of dried products: (**a**) macro-image of dried sample at 0% protein microgel concentration; (**b**) macro-image of dried sample at 4% protein microgel concentration; (**c**) macro-image of dried sample at 0% XG concentration; and (**d**) macro-image of dried sample at 1.2% XG concentration.

The microstructure of the dried samples with varying EWP microgels and XG concentrations are shown in Figure 4. Since the concentrations of EWP microgels and XG are clearly related to the properties of the emulsion, the lowest, middle, and highest concentrations were selected for more precise studies. As can be seen in the dried samples before shearing, the hydrophilic substance is in the continuous phase, while the oil is in the dispersed phase [8]. With an increase in EWP microgel concentration, the oil droplets gradually became smaller. When the EWP microgel concentration was less than 0.5%, it could not maintain the interface stability of the emulsions. Therefore, during the drying process, the oil droplets aggregated and formed large droplets, as seen in Figure 4a. The interfacial protein and the protein layer outside the droplets could effectively prevent the emulsion’s instability during the drying process with the increasing of the protein microgel concentration. In addition, the outer protein layer of the dried samples formed at a 4% protein microgel concentration was thicker than that formed at 2% because of the ‘greener layer’, which showed that when the adsorption of protein reached saturation, the excess protein gathered outside of the oil droplets to form a thicker protein film [45], or during the continuous phase. As shown in Figure 4d–f, when the concentration of XG increased, the distribution of oil droplets became more uniform, and the outer green layer of the oil droplets became thicker. In the emulsion system, the EWP microgel with the XG cooperatively maintained the stability of the oil droplets, and then still adhered to the outer layer of the oil droplets after the water was completely removed [28]. And excessive EWP or XG could coat the surface of the oil droplets, forming a thicker interfacial network. Or they existed in the continuous phase and stabilized the system. Meanwhile, as can be seen in the macro-pictures of the dried products in Figure A1, the addition of EWP microgels and XG had an obvious stabilizing effect. Oil leakage occurred in the dried samples without protein as an emulsifier or XG as a thickener. After drying, oil leakage was more pronounced in the sample with 0% EWP microgel concentration, possibly due to the lack of an emulsifier and difficulty in coating the oil droplets. When the concentration of XG was 0%, the particle size of the emulsion was large, the mechanical properties were weak, and the emulsion was unstable, which made it difficult to withstand the drying process.

#### 3.2.2. Stability of Oleogels

**Figure 5:**
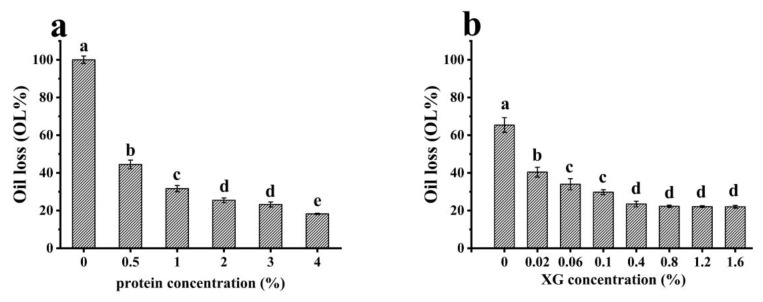
Effect of: (**a**) EWP; and (**b**) XG concentrations on oil loss (OL) value of oleogels. The error bars are the standard deviation of three repetitions. Different lowercase letters show significant differences (*p* < 0.05).

The stability of the oleogels was demonstrated by the amount of oil loss (OL%) (Figure 5). Lower oil-loss values revealed the better oil-binding capacity of the oleogels [46]. When the protein concentration was 0%, only XG was present, and the OL value of the oleogels was 100%. With the increase of EWP microgel concentrations, the OL value of the oleogels gradually decreased (*p* < 0.05), indicating that the adsorption of protein on the outer layer of the oil droplets helped to improve the oil-holding capacity of the oleogels. The result was consistent with a previous study, in which a sufficient amount of gelator was needed to cover the surface of the oil droplets and form the networked structure so that oil leakage was prevented [47]. Wijaya, et al. [48] also found that the oil loss of oleogels formed by sodium caseinate and alginate (SC-ALG) decreased as protein concentrations increased, and an increase in the proportion of protein was more critical in the production of physically stable oleogels. Moreover, with an increase in the protein concentration from 2% to 4%, the OL value of the oleogels also decreased, indicating that the thickness of the protein film adsorbed outside the oil droplets also contributed to the oil-holding capacity of the oleogels. A stronger interfacial structure helped to stabilize the droplets, leading to the improved stability of the oleogels [46]. In order to more clearly see the influence of XG concentration on OL, it was necessary to show the gap between the data, so data of some samples with different ratios were added. As shown in Figure 5b, with increasing concentrations of XG, the oil loss of oleogels gradually decreased. However, the OL value remained unchanged when the concentration of XG was larger than 0.4% (*p* > 0.05). The results indicated that the increase of XG to a certain level had little effect on the stability of oleogels. Previous studies have shown that the oil loss decreased with an increase in XG concentrations from 0.1 to 0.6%, while oil losses remained unchanged at higher concentrations [12]. The results indicated that the increase of XG did not obviously improve the OL of oleogels, which might also be related to the higher mechanical properties of the emulsion [28].

### 3.3. Oxidation Stability of Oleogels

**Figure 6:**
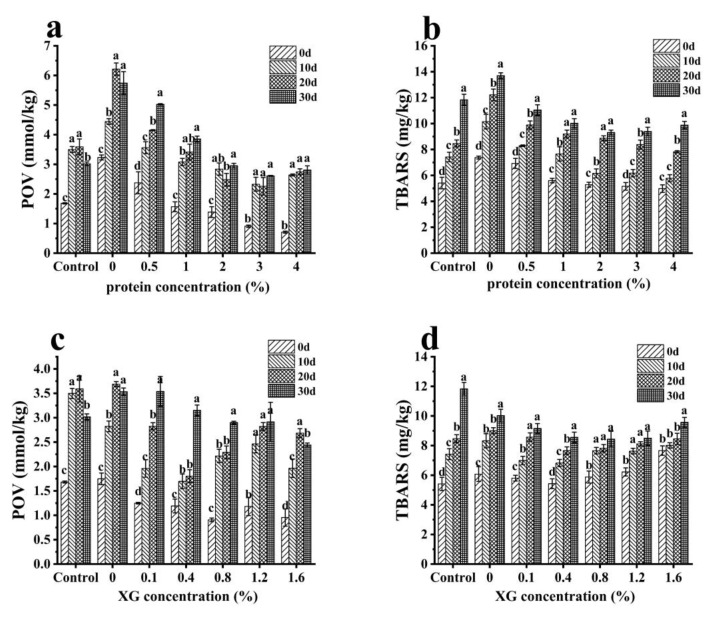
Effect of (**a**,**b**) EWP; and (**c**,**d**) XG concentrations on the peroxide value (POV; **a**,**c**) and thiobarbituric acid reactive substances (TBARS; **b**,**d**) of oleogels. The error bars are the standard deviation of three repetitions. Different lowercase letters show significant differences (*p* < 0.05).

Since oleogels were obtained through the drying process, the accelerated lipid oxidation that occurred during the drying process inhibits the application of oleogels in food systems [10]. Therefore, it is necessary to explore the oxidation stability of oleogels. Figure 6 shows the changes in POV and TBARS values of oleogels prepared with different concentrations of EWP microgels and XG when stored at 25 °C for 0, 10, 20 and 30 days. The control sample was vegetable oil dried in the oven at 60 °C for 24 h (the same drying conditions as oleogels). As shown in Figure 6a, the POV value of oleogels prepared with the 0% protein concentration was significantly higher than that of the control samples during storage. This might be because oil droplets are more likely to be exposed to oxygen/water in the preparation of the oleogels; thus the oil is prone to oxidation. When the protein concentration increased, the POV value of the oleogels decreased, and the POV value of the oleogels prepared with 2, 3 and 4% protein microgels was significantly lower than that of the control samples within 20 days. This was because, with the increase of protein concentration from 0 to 2%, the protein adsorbed outside the oil droplets, preventing the oil droplets from contacting oxygen and thus delaying the oxidation process as reported by Pan, et al. [49]. When the protein concentration was higher than 2%, the interface adsorption was saturated, the thick protein film formed by excessive protein adsorption on the surface of the droplets may have chelated metal ions and scavenged free radicals, thereby inhibiting the oxidation of oil, as described by Coupland et al. and Hu et al. [50,51]. As can be seen in Figure 6b, the TBARS values of oleogels with 0–1% protein concentration were higher than that of the control samples. At a low protein concentration, proteins could not completely encapsulate the oil droplets, and small oil droplets with large specific surface areas inevitably accelerated the process of oil oxidation. The TBARS values with a 0% protein concentration remained the highest during the 30 days, while the TBARS value of oleogels with increased protein concentrations decreased within 20 days. It is noteworthy that the TBARS values of all oleogels with protein (Samples with 0.5% to 4% protein concentration correspond 11.05, 10.02, 9.31, 9.39 and 9.88 mg/kg) were lower than the control (11.83 mg/kg) on the 30th day, even for samples with low protein concentrations (0.5% and 1%). Carina, et al. [52] also found that lower TBARS was associated with higher protein content, which may be related to the antioxidant properties of proteins. It may also have something to do with the fact that the protein acts as a physical barrier, protecting the oil from oxidation.

However, the increase in the XG concentration might have no significant positive effect on the inhibition of lipid oxidation in oleogels (Figure 6c,d). When the concentration of XG increased from 0 to 0.4%, the POV values had a decreasing trend. However, as the concentration of XG continued to increase, the POV values of the oleogels increased, even though the sample with 1.6% of XG concentration showed the lowest POV value on the 30th day. The TBARS values of oleogels with increased XG concentrations showed few changes, but oleogels with a 0–1.6% of XG concentration had lower values than the control on the 30th day. Moradabbasi, et al. [53] reported that the results showed no significant difference in the oxidation stability of both oleogels prepared with different XG concentrations (0.2% and 0.4%) and oil.

## 4. Conclusions

In this paper, a new kind of oleogel was prepared by the emulsion-template method using egg-white protein microgel particles as a gelator and xanthan gum as a thickener. The concentration of EWP microgel particles and XG played an important role in the properties of the emulsions and oleogels. When the protein concentration of the EWP microgel increased from 0–4% with a certain concentration of XG, a high concentration of protein tended to form an emulsion with better physical stability and higher mechanical strength, resulting in oleogels with a tighter network structure, better oil-binding capacity and the ability to prevent the oxidation of oils. When the concentration of XG was at 0–0.4% accompanied by a certain protein concentration, Ethe WP microgel combined with the XG effectively improved the properties of the emulsion and the stability of the oleogel. When the concentration of XG was at 0.4–1.6%, the excessive XG improved the viscosity and mechanical properties of the emulsion and blocked the aggregation and movement of the droplets, which was conducive to maintaining the physical stability of the emulsion. However, the increase in the concentration of XG (0.4–1.6%) had no significant improvement on the resistance to lipid oxidation of oleogels in this range. The results in this study could provide academic guidance for the preparation of oleogels by the emulsion-template method with protein and polysaccharide as oil-structuring agents.

## Figures and Tables

**Table 1:** Material ratio in EWP microgel concentration gradient experiment.

Amount/g in a 100 g Basis of Emulsion	0%	0.5%	1%	2%	3%	4%
EWP microgels	0	0.275	0.55	1.1	1.65	2.2
XG	0.18	0.18	0.18	0.18	0.18	0.18
Oil	30	30	30	30	30	30
Water	69.82	69.545	69.27	68.72	68.17	67.62

**Table 2:** Material ratio in XG concentration gradient experiment.

Amount/g in a 100 g Basis of Emulsion	0%	0.1%	0.4%	0.8%	1.2%	1.6%
EWP microgels	1.1	1.1	1.1	1.1	1.1	1.1
XG	0	0.0425	0.17	0.34	0.51	0.68
Oil	30	30	30	30	30	30
Water	68.9	68.8575	68.73	68.56	68.39	68.22

## Data Availability

The data presented in this study are available on request from the corresponding author.
